# Gene expression profile in colon cancer therapeutic resistance and its relationship with the tumor microenvironment

**DOI:** 10.3389/fbinf.2025.1674179

**Published:** 2025-10-29

**Authors:** Priscila Galvão Doria, Gisele Vieira Rocha, Vanessa Dybal Bertoni, Roberto de Souza Batista dos Santos, Mariana Araújo-Pereira, Clarissa Gurgel

**Affiliations:** 1 Gonçalo Moniz Institute, Oswaldo Cruz Foundation (IGM- FIOCRUZ/BA), Salvador, Brazil; 2 Department of Pathology and Foresinc Medicine, School of Medicine of the Federal University of Bahia, Salvador, Brazil; 3 Center for Biotechnology and Cell-Therapy, DO’R Research and Education Institute (IDOR), Salvador, Brazil; 4 Department of Propaedeutics, School of Dentristy of the Federal University of Bahia, Salvador, Brazil

**Keywords:** colon cancer, therapeutic resistance, gene expression profile, prognosis, biomarkers, computational biology, tumor microenvironment

## Abstract

**Introduction:**

Colon cancer is a common disease, treated with few chemotherapeutic agents with similar treatment sequencing despite its heterogeneity. A significant proportion of patients are diagnosed with metastasis, and resistance to antineoplastic drugs is associated with disease progression and therapeutic failure. It is known that the tumor microenvironment plays an essential role in cancer progression, contributing to processes that may be associated with therapeutic resistance mechanisms in colon cancer. In this study, we aim to identify a gene expression signature and its relationship with immune cell infiltration in colon cancer, contributing to the identification of potential resistance biomarkers.

**Methods:**

An *in silico* study was conducted using RNA-seq data from The Cancer Genome Atlas Program (TCGA) samples, subdivided into two groups (treatment-resistant and non-resistant), taking into account the molecular subgroups (CMS1, CMS2, CMS3, and CMS4). The following algorithms were used: i. *Limma* was applied to identify differentially expressed genes; ii. WGCNA was applied to construct co-expression networks; iii. CIBERSORT was applied to estimate the proportion of infiltrating immune cells; and iv. TIMER was applied to explore the relationship between core genes and immune cell content.

**Results:**

Twenty differentially expressed genes (DEGs) were found, with 18 related to the group considered resistant to oncologic treatment and presenting poorer overall survival. T CD4 memory resting cells and M0 and M2 macrophages were found in more significant proportions in the analyzed samples and more infiltrated in the tumor microenvironment, the higher the expression of some of these resistance DEGs. Additionally, these genes correlate with biological aspects of neuronal differentiation, axogenesis, and synaptic transmission.

**Conclusion:**

The gene expression signature suggests the presence of differentially expressed synaptic membrane genes, which may be involved in neuronal pathways that influence the tumor microenvironment, potentially serving as future biomarkers. Furthermore, the presence of M0 and M2 macrophages and T CD4 memory resting cells suggests a potential interaction that may play a role in therapeutic resistance.

## 1 Introduction

Colon cancer is the third most common cancer and the second leading cause of cancer-related death worldwide, with approximately 30% of patients diagnosed at an advanced stage and up to 50% developing metastatic disease, even when diagnosed at earlier stages (Sung et al., 2021; Malvezzi et al., 2018; Cervantes et al., 2023). Most patients with advanced disease have a median overall survival of approximately 37 months in the best prognostic scenario and a median progression-free survival of 10–13 months in first-line treatment and 4 months in second-line treatment, highlighting the impact of therapeutic resistance (Douillard et al., 2014; Heinemann et al., 2014; Cervantes et al., 2023; Shinozaki et al., 2021; Sobrero et al., 2008; Grothey et al., 2013; Mayer et al., 2015). It is widely recognised that colon tumors exhibit significant heterogeneity, both intra and intertumorally, as well as temporally and across metastatic sites (Morris and Strickler, 2021; Chen et al., 2023). Despite of this, most patients receive similar treatment sequences. Currently, no biomarkers are available in clinical practice to predict chemotherapy resistance.

Several mechanisms are associated with resistance to anticancer drugs, which may be linked to genetic alterations in tumor cells and patient-specific characteristics. These mechanisms range from poor drug absorption, rapid metabolism, and treatment intolerance to specificities of the tumor microenvironment, such as drug metabolism by non-tumor cells, blood supply, neovascularization, and how tumor cells interact (Pluen et al., 2001; Green, Frankel, and Kerbel, 1999; Gottesman, 2002). To date, research has predominantly focused on tumor cells in the search for biomarkers. However, tumors are influenced by molecular interactions with immunomodulatory functions within the tumor microenvironment. This immunomodulation is dynamically regulated by cell-to-cell interactions, soluble factors secreted by cells, extracellular matrix-mediated and microbiome interactions, all of which foster a pro-tumoral niche (Plundrich et al., 2022). Gene expression can be assessed through transcriptomic analyses of tumor samples to understand these processes better and identify potential strategies to block pro-tumoral stimuli (Hoadley et al., 2014).

Between 2012 and 2014, six groups proposed molecular subclassifications of colorectal cancer based on public gene expression databases, such as TCGA and GEO (Marisa et al., 2013; Sadanandam et al., 2013; Schlicker et al., 2012; Sousa et al., 2013; Budinska et al., 2013; Roepman et al., 2014). These efforts were unified in 2015, resulting in the consensus of four molecular subtypes: CMS1 (immune), CMS2 (canonical), CMS3 (metabolic), and CMS4 (mesenchymal) (Guinney et al., 2015). However, this subclassification is not yet used in clinical practice due to high costs, overlapping characteristics between subgroups, and the lack of direct correlation between each subgroup and treatment response patterns (Cohen et al., 2020). Therefore, it is valuable to explore the transcriptomic characteristics of these subtypes, considering patterns of resistance or sensitivity to chemotherapy, to translate the knowledge of CMS subgroups into more practical clinical markers, such as immunohistochemical or NGS-based markers. Furthermore, it is essential to identify specific treatment responses or resistance patterns.

This study aims to evaluate the gene expression profile and immune cell populations in colon cancer, considering treatment progression and molecular subgroups, to contribute to the identification of potential biomarkers of therapeutic resistance.

## 2 Materials and methods

**FIGURE 1:**
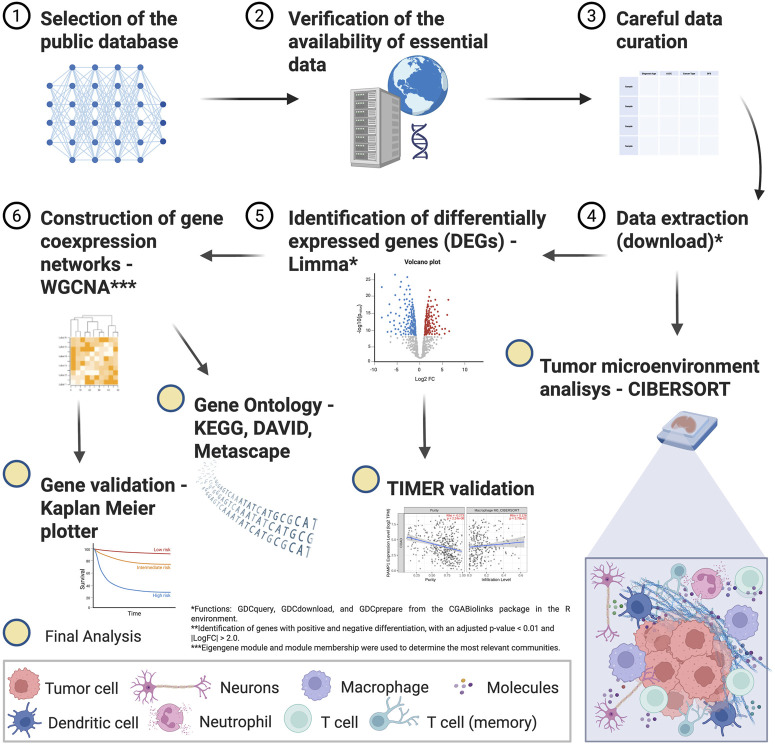
Flowchart of the *in silico* study methods.

The flowchart of the study steps is presented in Figure 1.

### 2.1 Data collection

Gene expression profiles and clinical data of patients with colon cancer were downloaded from the cBioPortal website (https://www.cbioportal.org). The following criteria were defined in the database selection tool: age >18, metastatic disease, available data on time to progression at first-line treatment, and molecular data with transcriptomics. (Supplementary Table S1). The data reflected the period of TCGA’s collection, during which standard first-line treatment was based solely on 5-FU–based chemotherapy, with limited variability in median progression-free survival, even with the addition of anti-EGFR or anti-VEGF agents. The 9-month cutoff was chosen to classify patients into non-resistant or resistant subgroups, based on the median progression-free survival reported in several pivotal trials of metastatic colorectal cancer treatment (Douillard et al., 2010; Douillard et al., 2014; Heinemann et al., 2014; Heinemann et al., 2021; Van Cutsem et al., 2009).

### 2.2 CMS subtype classification and deconvolution of immune cell profiles

RNA-seq data from 111 colorectal cancer samples, obtained from Bioconductor, were normalized using FPKM and mapped with the hg133plus2.db annotation, considering only protein-coding genes. Sample classification into CMS subtypes was performed using the CMScaller tool (version 2.0.1) (Eide et al., 2017).

Immune cell subtype characterization was conducted using CIBERSORT-X (cibersortx.stanford.edu) (Steen et al., 2020), which employs the LM22 gene signature matrix, consisting of 547 genes that accurately distinguish 22 immune cell types. After RNA-seq data normalization, immune profiles were obtained by calculating absolute scores. The Wilcoxon rank-sum test was used to compare immune cell proportions between resistant and non-resistant groups. To evaluate the association between DEG expression levels and immune cell infiltration, we used the TIMER platform, which is limited to data from the overall TCGA cohort and does not provide information restricted to metastatic cases. We considered that exploring these correlations in the entire TCGA dataset would provide a more biologically representative landscape, as a larger sample size increases the robustness of the analysis and may better capture the interactions between gene expression and the tumor microenvironment across different biological contexts of colorectal cancer. To assess the relationship between immune cell proportions and the expression of the most relevant DEGs, and to validate the findings obtained from TIMER, we performed correlation analyses using two complementary statistical methods: Spearman’s and Kendall’s correlation coefficients.

### 2.3 Identification of differentially expressed genes (DEGs)

Raw count and gene expression data were accessed using the R package *TCGABiolinks* (version 2.28.3) (installed through https://www.bioconductor.org). Genes in the matrix were annotated using information from the Affymetrix Human Genome U133 Plus 2.0 Array annotation data. We remove genes without annotation, and only those classified as protein-coding were retained for analysis. For genes with multiple spots, the one with the highest variance was selected. Differential gene expression analysis was performed by constructing a summarized object using the edgeR package (version 3.42.4). To ensure the inclusion of only relevantly expressed genes, those with counts per million (CPM) below the cutoff value (cutoff = 1) were excluded. The *Limma-voom* package (version 3.56.2) was used to analyze gene expression differences between non-resistant and resistant samples (Ritchie et al., 2015). Upregulated and downregulated DEGs were identified by filtering genes with an adjusted p-value <0.05 and |log2| fold change [LFC|] > 1.5.

### 2.4 Weighted gene co-expression network analysis

A co-expression network was built to investigate the correlation between expression and clinical outcomes of interest. DEGs were selected for weighted gene co-expression network analysis using the R package WGCNA (version 1.73) (Langfelder and Horvath, 2008). The gene expression matrix was converted to an adjacent matrix using Pearson’s correlation coefficient. The soft-thresholding power was determined using the *PickSoftThreshold* function to ensure a scale-free network. Then, the topological overlap matrix was calculated according to the corresponding soft-thresholding power (β = 3) (Supplementary Figure S1A). Hierarchical clustering was performed to identify the modules of densely interconnected genes (Supplementary Figure S1B). The correlation between the module eigengene (ME), which represents the first principal component of the module, and the clinical traits of breast cancer was calculated to identify clinically significant modules. Individual genes’ module membership (MM) values were used to screen for hub genes. Module membership (MM) was defined as the correlation between individual gene expression profiles and the ME of a given module. The initial gene validation comprised a comparative analysis of tumoral and non-tumoral relative gene expression and a survival investigation.

### 2.5 Pathway and functional enrichment analysis

The functions of the biological gene modules were investigated using Gene Ontology (GO) enrichment analysis. Functional annotation was complemented by the Kyoto Encyclopedia of Genes and Genomes (KEGG) database, which included genomic, chemical, functional, and metabolic information (Kanehisa et al., 2010). GO and KEGG analyses were performed using the DAVID (https://david-d. ncifcrf.gov/) database. The p < 0.05 value was estimated to consider the GO and the statistically significant functional enrichment.

### 2.6 Survival analysis

Initial survival analysis was performed using the R package for each DEG in the selected TCGA bank data.

Kaplan-Meier survival curves were generated to estimate overall survival in treatment-resistant and non-resistant groups. The log-rank test was used to assess differences between the survival distributions of these groups. The analysis was performed in the R programming environment, using the survival packages (version 3.8.3) to create and visualize the survival curves. Survival times were measured in months, and a p-value <0.05 was considered statistically significant.

## 3 Results

### 3.1 Identification of differentially expressed genes

**FIGURE 2:**
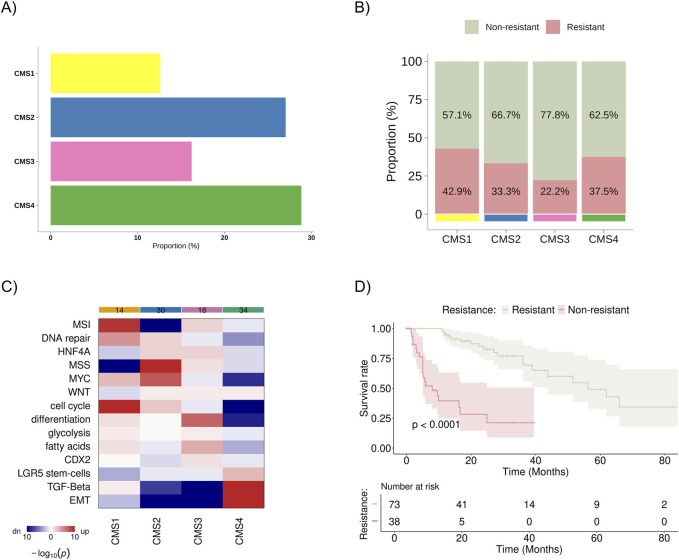
Distribution and characteristics of the study population sample. **(A)** Baseline distribution of consensus molecular subtypes (CMS1–CMS4) in the cohort **(B)** Proportion of patients classified as resistant (progression within 9 months of treatment) and non-resistant (progression after 9 months of treatment) within each CMS group, with values normalized to 100% across all subtypes. **(C)** Heatmap showing the results of gene set analysis based on mRNA sequencing, confirming the known characteristics of each CMS group. Color spectrum: red indicates upregulation, and blue indicates downregulation. **(D)** KM survival curve for overall survival between resistant and non-resistant groups. MSI, microsatellite instability; HNF4A, hepatocyte nuclear factor 4 alpha; MSS, microsatellite stability; MYC, pathway related to the c-Myc proto-oncogene; WNT, pathway related to the WNT protein-coding gene; CDX2, caudal-type homeobox 2; LGR5, leucine-rich repeat-containing G-protein coupled receptor 5; TGF-β, transforming growth factor beta; EMT, epithelial-mesenchymal transition.

For this study, the public database TCGA Firehose Legacy (COADREAD/20160128) and PanCancer Atlas available on cBioPortal were analyzed (Gao et al., 2013; Cerami et al., 2012), which included 1,232 patients with colon cancer. After excluding participants with missing data or duplicates, 111 patients with metastatic colon cancer remained for analysis. They were subdivided as follows: 73 patients were classified as non-resistant (disease progression after 9 months of initial treatment), and 38 were classified as resistant (disease progression within 9 months of initial treatment). Regarding CMS classification, 14, 30, 18, and 32 patients were included in CMS1, CMS2, CMS3, and CMS4, respectively (Figure 2A). These data were not available for 17 patients. No statistically significant difference between the proportion of resistant/non-resistant patient samples and the CMS groups was identified (Figure 2B). Still, higher percentage of resistant patients was observed in the CMS1 (42,9%) and CMS4 (37,5%) subgroups. The gene set analysis for each subgroup confirmed previously known characteristics: CMS1 was enriched for alterations in DNA repair pathways/microsatellite instability, MYC, and cell cycle pathways; CMS2 showed upregulation of MYC and cell cycle pathways and downregulation of microsatellite instability; CMS3 exhibited more significant upregulation of cellular differentiation and fatty acid pathways; and CMS4 was enriched for TGF-beta signaling pathways, TEM, and showed poor enrichment for differentiation pathways, cell cycle, MYC, and MSI (Figure 2C). In the Kaplan-Meier analysis, we observed a statistically significant difference in overall survival between the resistant and non-resistant patient groups (Figure 2D).

**FIGURE 3:**
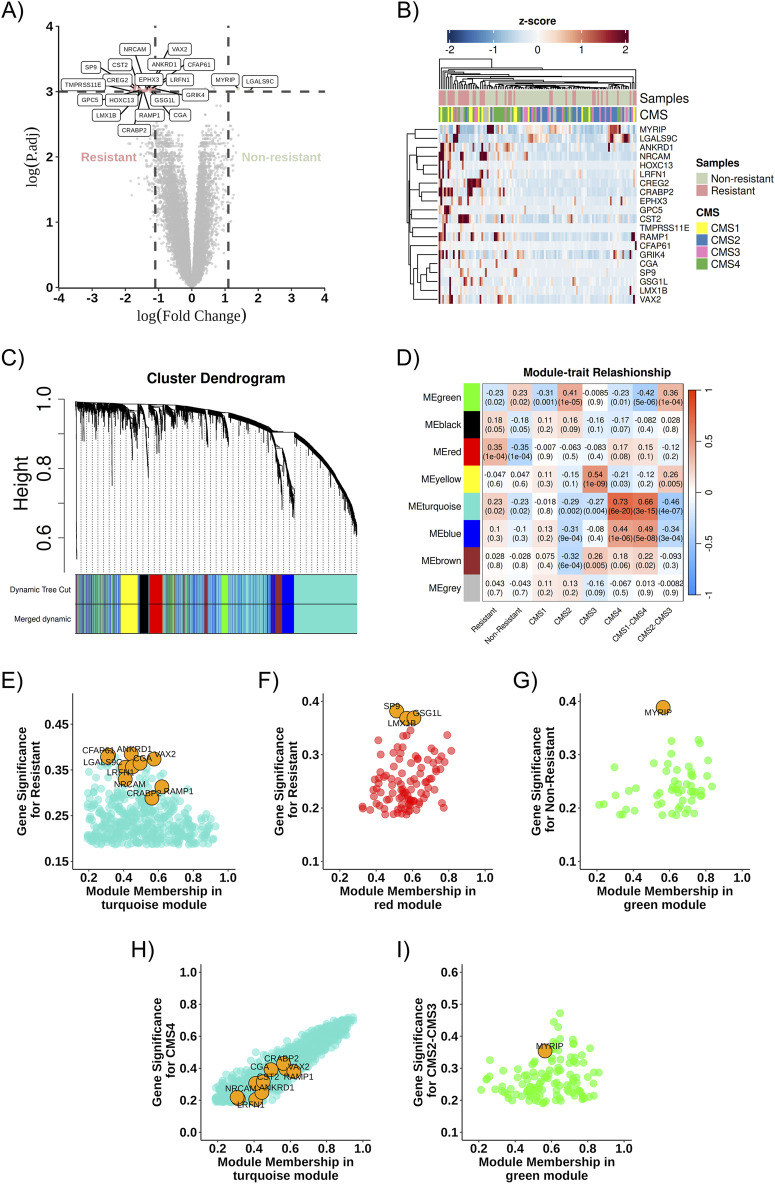
Co-expression network and DEGs. **(A)** Volcano plot highlighting the identified DEGs, comparing Resistant (left) and Non-Resistant (right) groups, logFC >2 and pValue <0.05. **(B)** Heatmap showing the identified DEGs and their relationship across groups (Non-Resistant, Resistant, and CMS1, CMS2, CMS3, CMS4); positively regulated genes are shown in red, and negatively regulated genes in blue. **(C)** Dendrogram of gene distribution in co-expression modules. **(D)** Heatmap representing the correlation strength between gene co-expression modules and the analysis categories. **(E)** Turquoise module about the resistance phenotype. **(F)** Red module about the resistance phenotype. **(G)** Green module about the non-resistant phenotype. **(H)** Turquoise module about the CMS4 phenotype. **(I)** Green module about the CMS2-CMS3 phenotype. The DEGs found in each community are highlighted in orange as potential cellular markers. DEG, differentially expressed gene; ME, Module eigengenes.

Through differential gene expression analysis using transcriptome data from RNA sequencing of the 111 samples described above, 20 differentially expressed genes were identified (Figures 3A,B) and a difference in gene expression profiles between the Resistant and Non-Resistant groups: 18 genes were upregulated in the therapeutic resistance group (CREG2, LRFN1, ANKRD1, GRIK4, CFAP61, NRCAM, CST2, SP9, VAX2, EPHX3, GPC5, CRABP2, RAMP1, LMX1B, CGA, TMPRSS11E, GSG1L, HOXC13), and two genes were upregulated in the treatment response group (MYRIP and LGALS9C) (Figure 3A).

### 3.2 Co-expression gene modules identification

The WGCNA algorithm was used to determine the DEGs co-expression gene network. The soft-threshold power (β) was set to three to ensure a scale-free network and the power fit index reached was 0.89 (Langfelder and Horvath, 2008). Gene co-expression network analysis resulted in eight modules, represented by green, black, red, yellow, turquoise, blue, brown, and gray colors (Figure 3C). The gene co-expression modules were correlated, and the molecular characteristics were classified according to resistance or non-resistance phenotypes and CMS subgroups: two modules (turquoise and red) were associated with the resistance phenotype. In contrast, one module (green) was more associated with the non-resistance phenotype. The turquoise module was more closely associated with CMS1 and CMS4 subgroups, whereas the green module was associated with CMS2 and CMS3 (Figure 3D).

### 3.3 Co-expression network and DEGs

After describing the most relevant communities in each phenotypic group, differentially expressed genes in the modules of interest for resistance and non-resistance were identified, and their connections to these modules were evaluated. Gene significance and MM aid in this connectivity assessment. As represented in Figures 3E–G, RAMP1, CRABP2, NRCAM, CST2, EPHX3, GPC5, LRFN1, CGA, VAX2, TMPRSS11E, HOXC13, CREG2 and CFAP61 correlates in the turquoise module, and GSG1L, LMX1B, and SP9 correlate in the red module, while MYRIP is found in the green module.

Moreover, some of these genes exhibit greater intramodular connectivity with the module. For example, RAMP1 showed a module membership value closest to 1 among the other identified DEGs, while ANKRD1 showed greater gene significance for resistance in the turquoise module (Figure 3E). Similarly, GSG1L has the highest module membership among the identified DEGs in the red module, and the DEGs found in the red module and the green module exhibit greater gene significance for resistance and non-resistance, respectively, compared to the other genes in the modules (Figures 3F,G).

Similarly, DEGs in the modules of interest about the CMS subtypes were sought. DEGs from the resistance group (CRABP2, RAMP1, VAX2, CREG2, NRCAM, ANKRD1, EPHX3, CST2, CGA, LRFN1, CFAP61, TMPRSS11E, HOXC13, GPC5, GRIK4) were observed in the turquoise module related to CMS1 and CMS4, while the non-resistance DEG (MYRIP) was found in the green module for CMS2 and CMS3, as represented in Figures 3H,I. The gene LGALS9C, more highly expressed in our non-resistant group, was found in the turquoise module associated with CMS1-CMS4; however, it had a low module membership value and low gene significance (Figure 3H).

### 3.4 Biological aspects related to gene communities (gene ontology)

**FIGURE 4:**
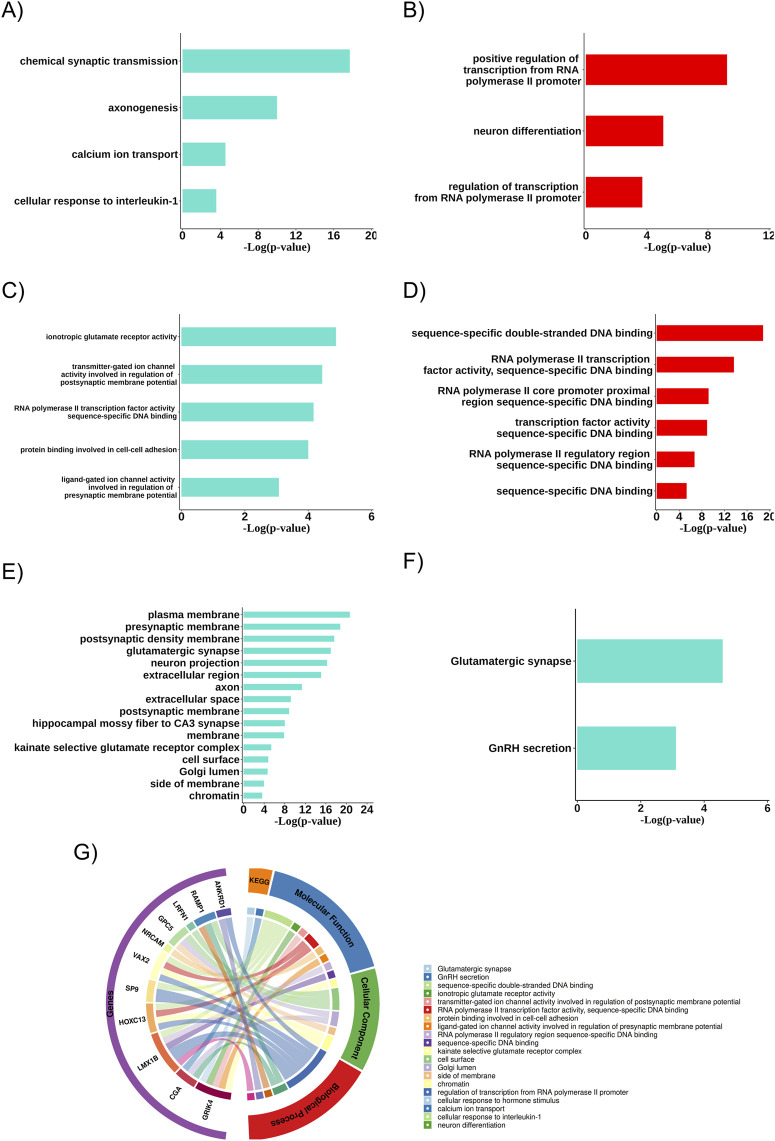
Main biological aspects related to the group of differentially expressed resistance genes. **(A)** Biological processes of the turquoise community. **(B)** Biological processes of the red community. **(C)** Molecular functions of the turquoise community. **(D)** Molecular functions of the red community. **(E)** Cellular components related to the resistance gene group of the turquoise community. **(F)** Key enriched pathways related to the resistance gene group of the turquoise community. **(G)** Biological aspects related to the differentially expressed (DEGs) resistance gene group.

The turquoise and red modules were analyzed about the biological systems associated with the genes due to their characteristics linked to the therapeutic resistance profile. In both cases, the biological processes with statistical significance (p < 0.05) and the presence of resistance-related DEGs were more associated with neuronal differentiation, axogenesis, synaptic transmission, transcriptional regulatory genes, calcium ion transport, and cellular response to IL-1 (Figures 4A,B). The biochemical activities (molecular functions) were also linked to ion channel activities involved in regulating pre-and post-synaptic membrane potential, ionotropic glutamate receptor activity, RNA polymerase II transcription factor activities, specific DNA binding, and protein binding involved in cell-cell adhesion (Figures 4C,D). Additionally, in the turquoise module, several relevant cellular components (the cellular locations where the related DEGs act) were identified, such as the plasma membrane, pre- and post-synaptic membrane, glutamatergic synapse, neuronal projection, extracellular region, axon, and extracellular space. We also found two pathways associated with the glutamatergic synapse and GnRH secretion (Figures 4E,F).

When analyzing the biological aspects related explicitly to the resistance-associated DEGs, an association with biological processes was observed, such as transcription regulation via RNA polymerase II promoter and cellular response to hormonal stimuli, with molecular functions related to double-stranded DNA sequence-specific binding and with cellular components such as the postsynaptic membrane (Figure 4G).

### 3.5 Analysis of cellular components, based on gene expression profile

**FIGURE 5:**
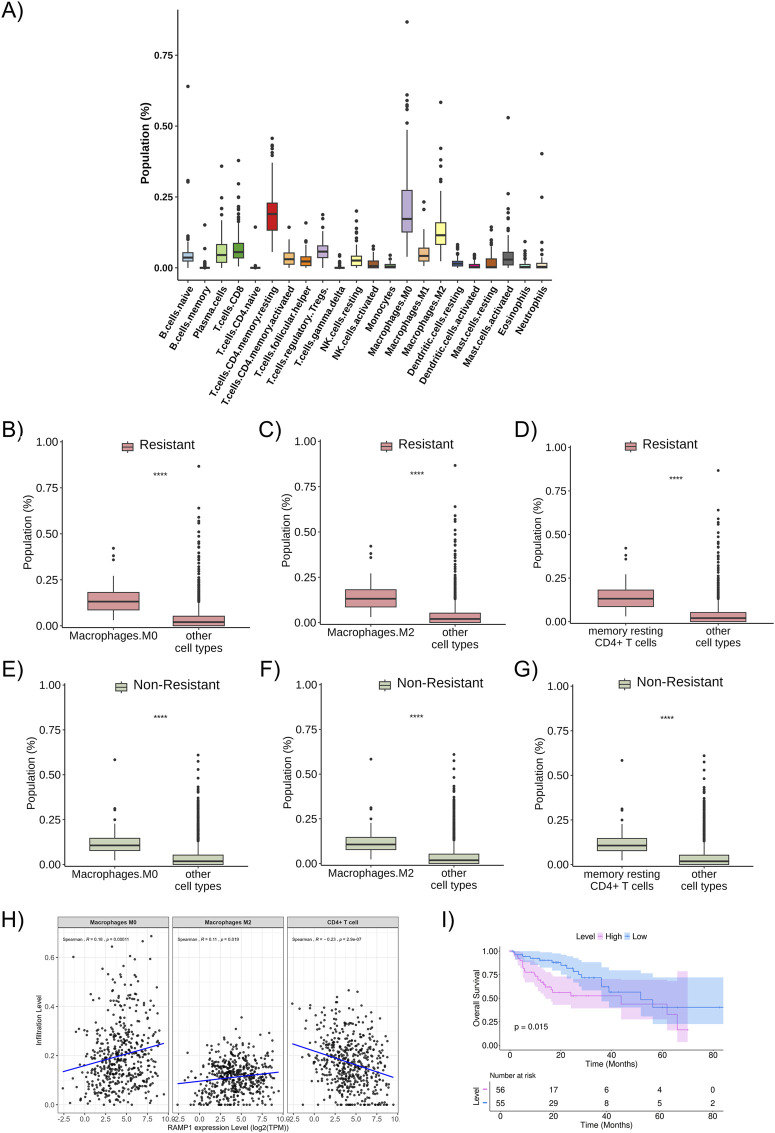
Proportion of immune cells in the studied population **(A)** Proportion and distribution of immune cell subtypes in the total studied population. **(B)** The proportion of M0 macrophages in the resistant patient group (red bars). **(C)** The proportion of M2 macrophages in the resistant patient group (red bars). **(D)** The proportion of resting memory CD4^+^ T cells in the resistant patient group (red bars). **(E)** The proportion of M0 macrophages in the non-resistant patient group (green bars). **(F)** The proportion of M2 macrophages in the non-resistant patient group (green bars). **(G)** The proportion of resting memory CD4^+^ T cells in the non-resistant patient group (green bars). **(H)** Relationship between the infiltration levels of M0 macrophages, M2 macrophages, and CD4^+^ T cells and the expression level of *RAMP1* (a resistance-related differentially expressed gene), considering the entire TCGA dataset for colon adenocarcinoma (and not only metastatic samples), with statistical correlation calculated using the Spearman method. **(I)** Kaplan-Meier curve of overall survival about the expression level of RAMP1 (resistance-related differentially expressed gene). ***: p < 0.0001.

The proportion of immune cells in the studied population (all metastatic patients included, regardless of resistance profile) was predicted using the CIBERSORT tool, with the most relevant cells in the sample: resting memory CD4^+^ T cells, M0 macrophages, and M2 macrophages (Figure 5A). These described cell fractions were present in significantly higher proportions in both the resistant and non-resistant groups (Figures 5B–G).

No difference was observed in the proportion of immune cells between the resistant and non-resistant groups. However, when studying the correlation between the cell fractions separately in the resistant and non-resistant groups, it was noted that there is a difference in the pattern of cellular interaction in the microenvironment of each group (Supplementary Figures S2A,B). In the non-resistant group, resting memory CD4^+^ T cells positively correlate with M1 macrophages and M2 macrophages; M0 macrophages positively correlate with gamma delta T cells, M2 macrophages, and activated mast cells; and M2 macrophages positively correlate with resting NK cells, resting memory CD4 T cells, M0 macrophages, M1 macrophages, and neutrophils, while negatively correlating with activated dendritic cells (p < 0.05). In the resistant group, resting memory CD4^+^ T cells positively correlate with naive B cells, activated memory CD4^+^ T cells, naive CD4^+^ T cells, and to a lesser extent with resting dendritic cells, while negatively correlating with activated NK cells, eosinophils, and monocytes; M0 macrophages positively correlate with gamma delta T cells, M2 macrophages, and negatively correlate with monocytes; and M2 macrophages positively correlate with gamma delta T cells, M0 macrophages, and M1 macrophages, and negatively correlate with memory B cells and resting NK cells (p < 0.05). Overall, these results indicate that the immune cell correlation networks differ between resistant and non-resistant tumors, suggesting variations in the organization of the tumor immune microenvironment.

### 3.6 Relationship between the expression of differentially expressed genes (DEGs) and immune cells and survival

To analyze the possible relationship between the expression levels of DEGs and immune cells, TIMER platform was used (data related to the total TCGA population, not only the patients with metastatic disease included in this study’s sample, as we considered that using the broader cohort could better represent the biological heterogeneity and immune context of colorectal cancer). A positive correlation was observed between RAMP1 expression levels and the infiltration of M0 and M2 macrophages, and a negative correlation with CD4^+^ T cells. Higher RAMP1 expression was associated with increased infiltration of M0 (p = 0.00011) and M2 macrophages (p = 0.019), and decreased infiltration of CD4^+^ T cells (p = 2.9 × 10^−7^) (Figure 5H). The correlations were further evaluated using Kendall methods, which showed consistent results (p = 1 × 10^−4^, p = 0.024, and p = 3.4 × 10^−7^) (Supplementary Figure S3). Similarly, a statistically significant positive correlation was found between M0 and M2 macrophages and higher expression levels of other resistance-related DEGs of interest, while a negative correlation was observed with resting memory CD4^+^ T cells (Supplementary Figure S4). In the analysis restricted to metastatic samples, a similar pattern was observed for macrophages, with higher RAMP1 expression correlating with increased infiltration of M0 macrophages in both resistant (p = 0.021) and non-resistant (p = 4.4 × 10^−5^) groups, and of M2 macrophages in resistant (p = 0.021) and non-resistant (p = 1.1 × 10^−7^) groups. A positive trend between RAMP1 expression and CD4^+^ T cells infiltration was noted in the metastatic cohort, but without statistical significance (resistant: p = 0.18; non-resistant: p = 0.25) (Supplementary Figure S5).

When analyzing the overall survival curves related to the differentially expressed resistance-associated genes (DEGs) identified in the study cohort, the overexpression of RAMP1 was associated with worse survival outcomes (Figure 5I), as were other DEGs such as SP9, LMX1B, HOXC13, GSG1L, GPC5, EPHX3, CGA, CFAP61, and ANKRD1 (Supplementary Figures S6A–I). Additional Kaplan–Meier curves for the remaining resistance-related DEGs are presented in (Supplementary Figures 7A–H).

## 4 Discussion

Gene expression profile and its relationship with immune cells in colon cancer tumor microenvironment was evaluated in this study to identify potential treatment resistance biomarkers. Our findings suggest that resistance-associated DEGs such as RAMP1, GSG1L, and GRIK4 may contribute to shaping the tumor microenvironment through interactions with M0/M2 macrophages and resting CD4^+^ memory T cells. The enrichment of biological processes related to neuronal differentiation, axonogenesis, and synaptic transmission further points to a role for altered cell–cell signaling and communication pathways in treatment resistance. Our results highlight molecular and cellular features that may underlie differential therapeutic responses in colon cancer.

Some of the identified DEGs are particularly relevant due to their roles in connectivity within interactive and coexpression networks, as well as their associated biological aspects and related functions. Genes such as RAMP1, LMX1B, GSG1L, HOXC13, CGA, ANKRD1 stand out. Among these, RAMP1 is particularly notable considering its involvement in biological processes and recent evidence highlighting its relationship with neuronal signaling and pro-tumor stimuli in the microenvironment (Xie et al., 2013; Balood et al., 2022).

High expression of RAMP1, that encodes the receptor for protein activity-modifying protein 1 (a coreceptor for certain G protein-coupled receptors on the plasma membrane), is associated with poorer oncological outcomes in osteosarcoma, likely as a secondary effect of alterations in the tumor microenvironment (L. Xie et al., 2023). Furthermore, studies suggest RAMP1 as a biomarker for tumorigenesis, impacting the MAP2KI (MEK1) signaling pathway (mitogen-activated protein kinase signaling pathway 2) (Logan et al., 2013) and its association with neuronal nociceptors and cancer disease progression (Balood et al., 2022). In our analysis, RAMP1 was differentially expressed in the chemotherapy-resistant group, interacting in a characteristic co-expression module for resistance (turquoise module) and related to the biological process of cellular response to hormonal stimuli. Higher levels of its expression correlate with decreased infiltration of CD4^+^ T cells and increased infiltration of M0 and M2 macrophages (considering colon adenocarcinoma samples regardless of clinical stage). In the analysis restricted to metastatic samples, no significant differences were observed in the interaction patterns between immune cells and DEGs. However, we observed potential differences in immune cell interactions suggesting that the tumor microenvironment of resistant tumors may be characterized by a less coordinated and potentially immunosuppressive cellular network, whereas non-resistant tumors display more balanced and possibly anti-tumor immune interactions. This observation reinforces the notion that resistance mechanisms may involve not only intrinsic tumor alterations but also distinct patterns of immune cell communication within the microenvironment. On the other hand, the absence of significant associations between immune cells and DEGs in the metastatic cohort may be related to the limited number of metastatic cases available, which could have reduced the statistical power to detect subtle immune–gene interactions. Moreover, the immune landscape within the tumor microenvironment is inherently heterogeneous, and such complexity is more accurately captured in analyses including a larger number of samples.

T cell activation requires three signals: the interaction between T cell receptors and antigens presented by antigen-presenting cells through MHC proteins; antigen-independent costimulatory signals derived from the interaction between CD28 on T cells and B7 family proteins (CD80) on antigen-presenting cells; and stimulatory cytokines concentrated at the immune synapse (Meissner et al., 2017; Sadelain et al., 2003). The formation of immune synapses facilitates tight intercellular communication, enhancing cytokine-mediated signaling (Dustin, 2014; Xie et al., 2013). M2 macrophages are typically anti-inflammatory, characterized by a poor capacity to present antigens, which leads to immunosuppressive effects and promotes cell proliferation, tissue repair, angiogenesis, and the release of immunosuppressive molecules in the tumor microenvironment, such as IL-10, TGF-β, and HLA-G (Allavena et al., 2008; Komohara et al., 2016). Moreover, tumor-associated macrophages (particularly the M2 subtype) have been shown to express higher levels of glial cell line–derived neurotrophic factor in pancreatic cancer compared to other macrophage subtypes (Cavel et al., 2012), and M2 macrophages express molecules that influence neoplastic proliferation through fibroblast growth factors (FGF) and epidermal growth factors (EGF) (P Allavena and Mantovani, 2012; Mitsudomi and Yatabe, 2010; Turner and Grose, 2010).

The colon has both intrinsic autonomic innervation (enteric nervous system) and extrinsic innervation (fibers from the vagus nerve and splanchnic nervous system). Preclinical studies have demonstrated that denervation of the enteric nervous system decreases neoplastic proliferation (Kannen et al., 2015; Vespúcio et al., 2008). Additionally, neurotrophic factors activate the MAPK/ERK signaling pathway, promoting metastasis (Lei et al., 2022). This pathway is known to be implicated in the pathophysiology of colorectal cancer with the BRAF V600E mutation. Furthermore, most cells present in the tumor microenvironment, such as cancerous cells, endothelial cells, fibroblasts, and immune cells, have receptors for neurotransmitters and are found around tumor microenvironment innervation (Wang et al., 2024). Therefore, interactions among a variety of cell types near tumor-associated nerves interfere with the local response to sympathetic or parasympathetic stimuli, as well as to neurotransmitters such as catecholamines and acetylcholine, respectively (Boilly et al., 2017; Zahalka and Frenette, 2020). The numerous interactions between tumor cells and the various cells and molecules in the tumor microenvironment are likely responsible for the complexity of oncological processes. In our study, several differentially expressed genes identified have neuronal functions and are localized to synaptic membranes or axons. Synaptic structures regulate cell–cell communication, information processing, and storage, potentially mediating interactions between molecular targets and influencing responses to targeted therapies or even immunotherapy (Alekseenko et al., 2020). Collectively, this literature supports the concept that neural systems may interact with local immune cells and, in line with our analysis, may contribute to therapeutic resistance in colorectal cancer. Nevertheless, our study is based on omics analyses and highlights potential associations rather than definitive mechanistic pathways.

In conclusion, we conducted an integrated *in silico* analysis to identify differentially expressed genes involved in a therapeutic resistance profile in samples from patients with metastatic colon cancer. A gene signature was identified with nine genes (RAMP1, LMX1B, GSG1L, HOXC13, CGA, ANKRD1, GRIK4, NRCAM e LRFN1) that suggests an association with neuronal pathway processes, influencing the tumor microenvironment and conferring therapeutic resistance, with particular emphasis on gene RAMP1. This signature may prove to be prognostically useful and with potential therapeutic targets in precision medicine. Further validation studies are necessary, focusing on investigating biological functions and biomarkers with practical applications.

## Data Availability

The original contributions presented in the study are included in the article/Supplementary Material, further inquiries can be directed to the corresponding authors.
