# Supplementary material for: Gene expression profile in colon cancer therapeutic resistance and its relationship with the tumor microenvironment
Source: Front Bioinform. 2025 Oct 29;5:1674179. doi: 10.3389/fbinf.2025.1674179 (PMC12604976; doi:10.3389/fbinf.2025.1674179)
Supplement: Supplementary file 1 [file Supplementaryfile1.docx]

***Supplementary Material***

# Supplementary Figures


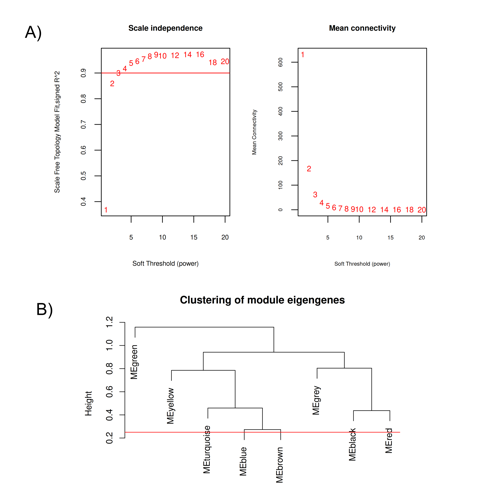


**Supplementary Figure 1.** Construction of the WGCNA network. (A) Determination of the soft-threshold power for constructing a scale-free network. (B) Dendrogram for module detection.


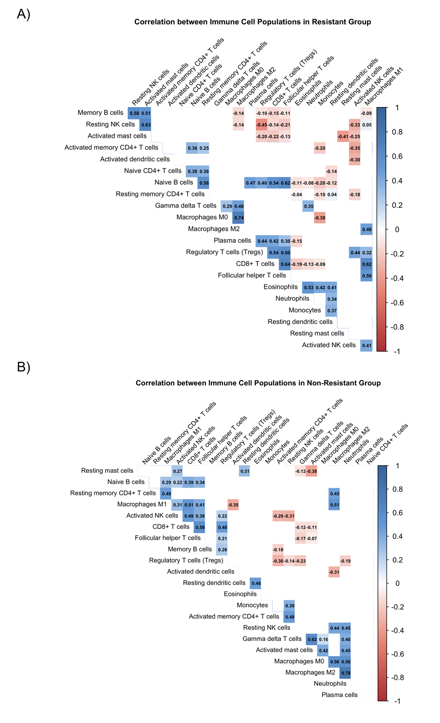


**Supplementary Figure 2.** Correlation between immune cell populations estimated by the CIBERSORT algorithm. (A) Correlation among immune cells in the resistant group. (B) Correlation among immune cells in the non-resistant group.


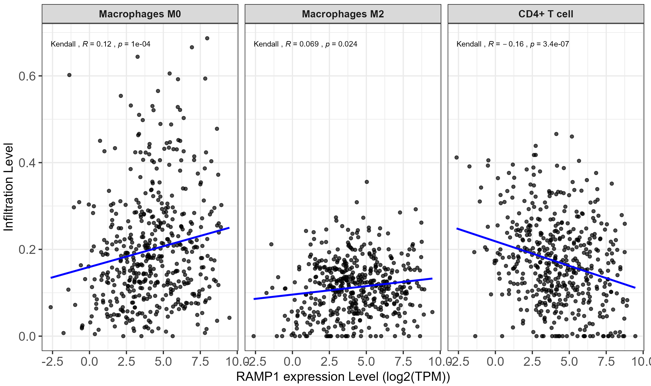


**Supplementary Figure 3.** Correlation between immune cell infiltration (M0 macrophages, M2 macrophages, and CD4⁺ T cells) and RAMP1 expression levels, calculated using Kendall’s rank correlation coefficient.


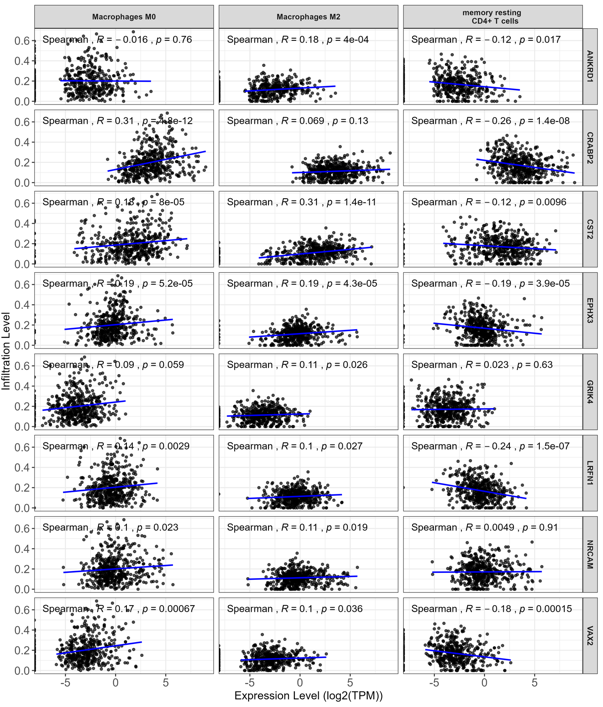


**Supplementary Figure 4.** Correlation between immune cell infiltration (M0 macrophages, M2 macrophages, and CD4⁺ T cells) and the expression levels of other resistance-related DEGs of interest, calculated using Spearman’s rank correlation coefficient.


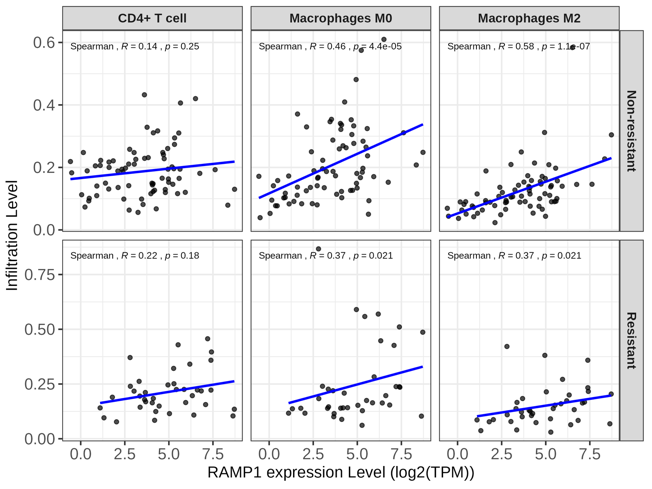


**Supplementary Figure 5.** Correlation between immune cell infiltration (CD4⁺ T cells, M0 macrophages, and M2 macrophages) and RAMP1 expression levels in the resistant and non-resistant groups, calculated using Spearman’s rank correlation coefficient.


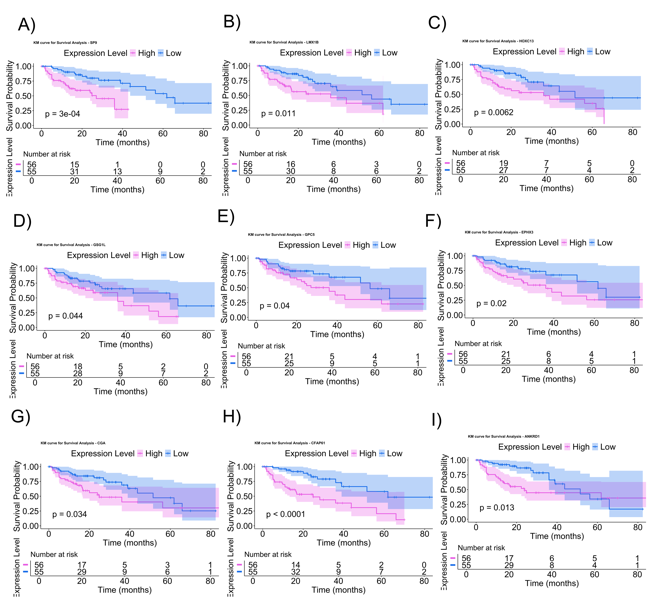


**Supplementary Figure 6.** Statistically significant Kaplan–Meier survival curves for resistance-related DEGs. Each panel shows the association between gene expression level and overall survival in our cohort. (A) *SP9*: higher expression associated with worse overall survival. (B) *LMX1B*: higher expression associated with worse overall survival. (C) *HOXC13*: higher expression associated with worse overall survival. (D) *GSG1L*: higher expression associated with worse overall survival. (E) *GPC5*: higher expression associated with worse overall survival. (F) *EPHX3*: higher expression associated with worse overall survival. (G) *CGA*: higher expression associated with worse overall survival. (H) *CFAP61*: higher expression associated with worse overall survival. (I) *ANKRD1*: higher expression associated with worse overall survival.

**
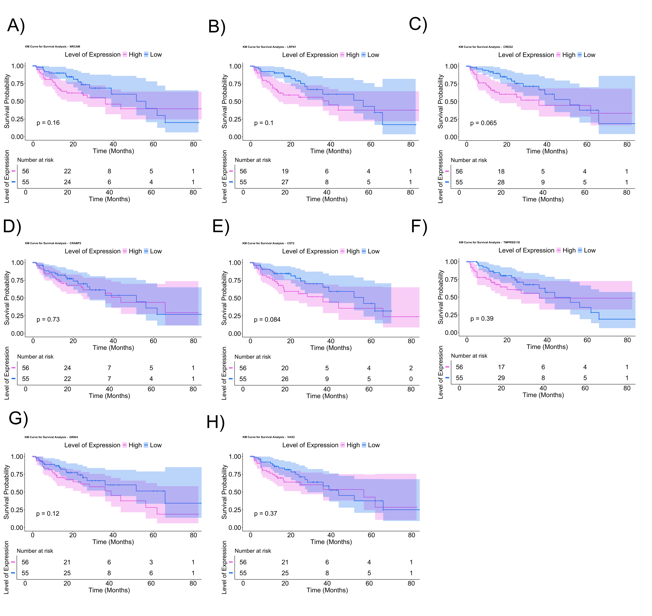
**

**Supplementary Figure 7.** Non-statistically significant Kaplan–Meier overall survival curves for other resistance-related DEGs. Each panel shows the association between gene expression level and overall survival in our cohort. (A) NRCAM: no significant difference in overall survival according to expression levels. (B) LRFN1: no significant difference in overall survival according to expression levels. (C) CREG2: no significant difference in overall survival according to expression levels. (D) CRABP2: no significant difference in overall survival according to expression levels. (E) CST2: no significant difference in overall survival according to expression levels. (F) TMPRSS11E: no significant difference in overall survival according to expression levels. (G) GRIK4: no significant difference in overall survival according to expression levels. (H) VAX2: no significant difference in overall survival according to expression levels.
